# Ague and Quinine

**Published:** 1882-06

**Authors:** 


### AGUE AND QUININE.

An animated discussion was going on some time ago in the journals as to the curability of the "Ague" by dynamized homœopathic remedies.

It will be necessary before discussing this question to premise our remarks by reminding the profession that "Ague" is a very peculiar form of disease. That its most virulent types are seen nowhere save at or near *each practitioner's residence!* Each physician considers his cases the worst that exists. Thus, a Western physician will consider Eastern "chills and fever" a mere nothing to cure; while the Eastern man laughs at the Western fever; and so it goes. This peculiarity materially lessens one's ability to cope successfully with this hydra-headed monster, as he has no authorities from whom he can garner experience; no authors, whose *ipse dixits* may be authoritative. Even the law of the similars, though all-embracing, does not seem to embrace this disease. It evidently comes under the head of "supplementary and auxiliary principles," but as these have not, *as yet*, been defined, they cannot assist us here.

The character of the disputants in this case is also well worthy of a passing notice, as they may throw some light on the question at issue. For the same difference will be found here, as in other cases, where the healing power of homœopathic medicines is disputed. On one side we find men who do *not* practice pure Homœopathy (hence they are not able or fit to judge of its powers). These are the men who deny that homœopathic medicines can cure "intermittent." On the other side, we find men who *do* practice Homœopathy as the law directs. These are the men who cure it. Whose testimony shall we believe?

No one, free from an asylum, would expect to cure any disease homœopathically unless he practiced Homœopathy. This we propose to prove these doubting Thomases do not do. We shall judge them by their own testimony, a fair proceeding, surely.

In the *Medical Advance* for August, 1880, a correspondent writes:

"If I understand Homœopathy, we are to prescribe the remedy which shall correspond to the totality of the symptoms. If any one who has had much experience in treating malarial diseases will carefully read the incomplete proving even of *Quinine* as given in Pereira's or any other allopathic materia medica, he will there see almost a perfect picture of the malarial fevers; consequently [?] he who gives *Quinine* in malarial fevers is prescribing according to the homœopathic law." * * * "Now I do not wish to be understood as saying that *Quinine* is homœopathic to all cases of chills and fever that a physician may meet, but that it is to the great majority, and clinical experience has proven that it will cure when we have the indications for its use." * * * "Now, if *Quinine* is homœopathic to ague, and *it is to the great majority of cases*,* why not give it and cure your patient, rather than ransack the materia medica to find some other remedy that might cure? Then, as true physicians, let us rise above that narrow plane, on which some of our school would have us stand, and be homœopathists in the highest sense of the term."

We have quoted at length from this writer, that his error—an error common to so many—may be fully appreciated, which is the lack of a full understanding of the Law of the Similars. His assertion that we should prescribe solely on "the totality of the symptoms," is certainly correct. But does he ever do it? We fear not; for if he did, he would never speak of a remedy as "homœopathic to ague." There is no remedy "homœopathic to ague," or to pneumonia, or diphtheria, or to any *disease.* Such an idea is as old as Sydenham and as lifeless. Every homœopathic remedy is a specific, a sure and certain specific for *its symptoms* and for nothing else.

To find the specific for a group of symptoms we must individualize closely. We must select not *any* similar, but *the most* similar remedy, *i. e.*, the *simillimum.* This is often tedious; it is very much easier to give quinine and break the chill "than to ransack the materia medica" to find the true curative remedy. In "ague" districts, as elsewhere, laziness sometimes conquers, and quinine is given, to the detriment of the patient and of Homœopathy.

To show how complete is our materia medica in the therapeutics of this disease, and also how necessary it is to strictly individualize each case, it may be stated that there are in Bœnninghausen's work on "Intermittent Fever," over one hundred drugs given, which have symptoms of intermittent fever. Most of these drugs have, in varying degrees, the three stages of chill, heat and sweat, but *no two* have them in *exactly* the same way. The variance may be as to the time of occurrence, as, for instance, in Podophyllum, 7 A. M. is

---

* We italicize these few words and beg they will be contrasted with the letter on "Malaria Mania" quoted at the end of this article.

the characteristic hour, or as in Cactus g., 11 A. M. (and 11 P. M.); while Arsenic and Cinchona have no special hour. Again, the variance may be as to amount of fever, the manner of its commencing or its concomitants. Thus Arsenic or Veratrum may have no heat, while Aconite or Bryonia has much heat. Yet, though these two latter remedies are similar as to amount of heat, they differ widely as to their restlessness; Aconite being very restless, and Bryonia the reverse.

Again, one remedy, like Ignatia, may be improved by external warmth, while another, as Ipecac, the chill may be increased by external covering. As a further illustration, taking the one symptom, "aversion to food," we find Arnica has this symptom during chill and fever, but not during sweat. Cyclamen has it during fever and sweat, not during chill; while Stramonium and Veratrum have it only during sweat. Baryta, Bell., Calc., Cicuta v., Cocculus and others, have it only during the fever. Thus we see how remedies differ even in one common symptom.

Examples of individualization of drugs could be indefinitely extended; these few, however, suffice to show how easy it can be done if one chooses to carefully study his case. As Hahnemann says, the most important part of the work is done when the physician has carefully written out his case. In no disease is this more useful than in intermittent fever, where the different stages, their duration, amount and concomitants, should be so carefully compared. A case once written out is easily compared with the materia medica; the more symptoms the easier. Yet so complete is our art that even the lack of symptoms is no bar, for the mere fact that a case is not fully developed is itself an indication for a remedy, as for instance, Arsenic.

Choose the simillimum remedy, give it high, in single dose (or in few quickly-repeated doses in water, which is often very beneficial), and await its action; give it time and never repeat dose, or worse, change the remedy, on an *improving* condition; do these things and we will cure our cases—*wherever found.*

A few words more and we are done with "Ague." The gentleman quoted also says: "Many claim that Quinine does not cure but suppresses the chill, and that after a time it will return again and then will not yield to Quinine. Now, according to my observation, the chill will not return any quicker when broken by Quinine than when broken by any other remedy." To this we reply, that a chill

"broken" is not cured, and one "broken" by one remedy is as bad treatment as one broken by another; a "break" or suppression differs from a cure in this very particular, that one returns, the other does not. Homœopathy never "breaks" chills, it cures them.

The doctor's experience, with his peculiar "Ague," differs widely from that of the allopaths in treating malarial diseases, for they find that chills *do* return after being "broken" by Quinine, and that they *do not* then yield readily to Quinine. And he seems much more successful, with his Quinine, than the "Regular," for Aitkin (vol. I, p. 591) says: "It is useless to attempt to cure intermittent fever [*with Quinine?*] if the sufferer is permitted to remain within the sphere of malarial influences, or even in those geographical latitudes which are said to be peculiarly malarial."

The same author also remarks, "the treatment varies, in a great degree, with the complications [*symptoms?*] of the disease." The allopath believes they must vary their treatment according to the "complications of the disease," yet our homœopathic friend declares one remedy will cure the large majority of agues! This indiscriminate exhibition of Quinine for every *malarious* trouble is nothing new; in fact, it has been done ever since the bark was discovered, in 1640—for 240 years—by the allopaths, who acknowledge it does not cure. They use it, as they have nothing better; they very sensibly do the best they can; but for one who, by pretending to be a homœopath, ought to know better, its use is simply criminal. The story of the abuse of Quinine by the "Regulars" is well told in the letter we print below. This abuse of Quinine in these days almost equals their former abuse of mercury, and, like that remedy, its popularity will some day die out, only to be superseded by another favorite; such being the history of a materia medica not founded on *law*.

---

The following letter from an allopath may possibly give such homœopaths, as the above-quoted gentleman, some new ideas on "Quinine:"

MALARIAL MANIA.

"*To the Editor of The New York Medical Record:*

"SIR: In a recent issue of *The Medical Record* I notice a correspondent has ventured to deny that malaria is the cause of all diseases to which humanity is heir. I am glad to know that one member of the profession has the brain to see and the courage to talk against this assumption of human ignorance. When I commenced the practice of medicine, thirty-five years ago, the liver was the only organ noticed in the human body, and calomel the only remedy. Now malaria is the source of all our woes and quinine the antidote.

"Being an old doctor, I am frequently called in consultation by the younger members of the profession, and for the last ten years I cannot call to mind a single case of any disease, from a stone bruise to a broken neck, in which the doctor had not given quinine. These doctors tell me the books recommend us to do so and so. Before a man writes a book he is just a common doctor like the balance of us; but as soon as he gravely writes his opinions down in a book they become as a voice of inspiration.

"Not long since I read an article at one of our county medical societies against the use of quinine in pneumonia. I said that if there was no more quinine used in the treatment of disease than was necessary, that quinine would not be worth a dollar an ounce. Well, my brethren said I was a heretic.

"There has been an epidemic here, during the last few days, among children. They had a fever, attended with a cough, and the disease ended in about seven days.

"My professional brethren all gave quinine, they said it was malaria, and the patients all got well. I could neither see nor smell the malaria, so I gave no quinine, and my patients all recovered also. Of course it was all malaria.

"There has been in this vicinity an epidemic of typhoid fever, or, as it is now fashionably called, typho-malarial fever. Some gave quinine from alpha to omega, but with no apparent benefit. It neither abated the violence, nor shortened the duration in the slightest degree in any case.

"The doctors who gave it said it was recommended in certain books, and they considered it their Christian duty to prescribe it. I was called to one of these cases on the ninth day of the fever, the case was a girl of fourteen. She was most inveterately delirious.

"The physician in attendance had give this beautiful maiden twenty grains of quinine per diem, for nine long, dreary days, and the stuff would neither act antipyretical or vaso-motorically!

"This doctor said the girl was 'pizened' with malaria, and he wanted to continue the quinine.

"The spleen has now superseded the liver. We have all become leucocythemic instead of bilious.

"I am living where malaria is supposed to reign supreme.

> "Where the crocodile croaks,
>   And the bullfrogs enchant;
> Where the pond-lilies bloom,
>   And the lizards do haunt.
> Where the goose and the duck
>   Forever do quake,
> As they dip their broad bills
>   In the mud of the lake.

"But, inasmuch as this malarial phantom does not put in his appearance unless the days are hot and the nights cool, I suppose I may be excused for not following the 'traditions of the elders.'" E. J. L.

